# Lysophosphatidic Acid-Activated Calcium Signaling Is Elevated in Red Cells from Sickle Cell Disease Patients

**DOI:** 10.3390/cells10020456

**Published:** 2021-02-20

**Authors:** Jue Wang, Laura Hertz, Sandra Ruppenthal, Wassim El Nemer, Philippe Connes, Jeroen S. Goede, Anna Bogdanova, Lutz Birnbaumer, Lars Kaestner

**Affiliations:** 1 Department of Cellular and Molecular Biology, The University of Texas Health Science Center at Tyler, Tyler, TX 75708, USA; anjwan@googlemail.com; 2 Theoretical Medicine and Biosciences, Saarland University, 66421 Homburg, Germany; laurahertz@gmx.de; 3 Experimental Physics, Dynamics of Fluids, Saarland University, 66123 Saarbrücken, Germany; slruppenthal@gmx.de; 4 Gynaecology, Obstetrics and Reproductive Medicine, Saarland University Hospital, 66421 Homburg, Germany; 5 Etablissement Français du Sang PACA-Corse, Aix Marseille Université, EFS, CNRS, ADES, 13005 Marseille, France; wassim.el-nemer@efs.sante.fr; 6 Laboratoire d’Excellence GR-Ex, 75015 Paris, France; pconnes@yahoo.fr; 7 Laboratory LIBM EA7424, Vascular Biology and Red Blood Cell Teal, University Claude Bernard Lyon 1, 69008 Lyon, France; 8 Division of Oncology and Hematology, Kantonsspital Winterthur, CH-8401 Winterthur, Switzerland; Jeroen.goede@ksw.ch; 9 Red Blood Cell Research Group, Institute of Veterinary Physiology, University of Zürich, CH-8057 Zürich, Switzerland; annab@access.uzh.ch; 10 Institute of Biomedical Research (BIOMED), Catholic University of Argentina, C1107AFF Buenos Aires, Argentina; birnbau1@gmail.com; 11 Laboratory of Neurobiology, National Institute of Environmental Health Sciences, Research Triangle Park, NC 27709, USA

**Keywords:** erythrocytes, sickle cell disease, LPA receptor, G protein signaling, transgenic mice, protein kinase Cα, MAP kinase, TRPC6, Ca_V_2.1, Gárdos channel

## Abstract

(1) Background: It is known that sickle cells contain a higher amount of Ca^2+^ compared to healthy red blood cells (RBCs). The increased Ca^2+^ is associated with the most severe symptom of sickle cell disease (SCD), the vaso-occlusive crisis (VOC). The Ca^2+^ entry pathway received the name of P_sickle_ but its molecular identity remains only partly resolved. We aimed to map the involved Ca^2+^ signaling to provide putative pharmacological targets for treatment. (2) Methods: The main technique applied was Ca^2+^ imaging of RBCs from healthy donors, SCD patients and a number of transgenic mouse models in comparison to wild-type mice. Life-cell Ca^2+^ imaging was applied to monitor responses to pharmacological targeting of the elements of signaling cascades. Infection as a trigger of VOC was imitated by stimulation of RBCs with lysophosphatidic acid (LPA). These measurements were complemented with biochemical assays. (3) Results: Ca^2+^ entry into SCD RBCs in response to LPA stimulation exceeded that of healthy donors. LPA receptor 4 levels were increased in SCD RBCs. Their activation was followed by the activation of G_i_ protein, which in turn triggered opening of TRPC6 and Ca_V_2.1 channels via a protein kinase Cα and a MAP kinase pathway, respectively. (4) Conclusions: We found a new Ca^2+^ signaling cascade that is increased in SCD patients and identified new pharmacological targets that might be promising in addressing the most severe symptom of SCD, the VOC.

## 1. Introduction

In sickle cell disease (SCD) patients’ red blood cells (RBCs), the Ca^2+^ uptake is increased to approximately 300 µmol/(l_cells_h) during vaso-occlusive crisis (VOC), which is approximately 6-fold higher than in healthy RBCs [1,2]. This Ca^2+^ overload is likely to be mechanistically involved in the generation of the VOC, e.g., favoring RBC dehydration that promotes crystallization of deoxyHbS. However, the deoxygenation of HbS alone is insufficient to explain the erratic nature of the crises [3]. Dehydration occurs due to Gárdos channel (Ca^2+^-activated K^+^ channel, KCNN4, K_Ca_3.1, hSK4) activation and the following K^+^ loss increases the mean cellular hemoglobin concentration (MCHC) and promotes HbS aggregate formation. Furthermore, Ca^2+^ overload promotes phosphatidylserine externalization already in healthy RBCs [4] but in particular in RBCs of SCD patients [5], and as such increases the aggregability of RBCs [6] and their intercellular adherence [7]. Thus, the increased Ca^2+^ may promote the perturbation of microcirculation with participation of even non-deoxygenated cells, independent of dehydration. This would increase RBC transit time in poorly oxygenated vascular areas and further increase the risks for VOC.

The Ca^2+^ increase was named P_sickle_ due to its conductive nature but without any knowledge about its molecular identity [8,9]. However, in recent years, it became obvious that more than one ion channel is involved in P_sickle_ [10,11]. One direct Ca^2+^ entry pathway, which is elevated in SCD RBCs, was shown to be the NMDA receptor [12,13]. A recent clinical trial with memantine, an antagonist of the NMDA receptor, showed promising results [14,15]. Here we provide evidence for a further player, lysophosphatidic acid (LPA), that activates a signaling cascade, leading to increased intracellular Ca^2+^ and this Ca^2+^ entry pathway is upregulated in RBCs of SCD patients. LPA-induced Ca^2+^ entry into RBCs was previously shown and believed to be channel mediated [16,17].

Whereas pharmacological studies pointed to the Ca_V_2.1 channel [17,18], patch-clamp measurements of channel activity gave rise to an alternative hypothesis that, instead, a non-selective cation channel might cause the Ca^2+^ influx [19,20]. Although LPA-induced Ca^2+^ influx was phenomenologically characterized [21,22,23], very little is known about the signaling pathways resulting in LPA-dependent ion channel activation. It is known that LPA activates protein kinase Cα (PKCα) [18,21,24], which is among the conventional and novel PKC isoforms and, according to current knowledge, the only abundant variant occurring in RBCs [25]. However, a putative interaction between PKC and the Ca_V_2.1 channel is rather controversial [18,24,26]. Therefore, we aimed to further elucidate the signaling pathway from LPA stimulation of RBCs to the Ca^2+^ entry mechanisms in healthy and SCD RBCs.

## 2. Materials and Methods

### 2.1. Preparation of Human and Mouse Blood Samples

Experiments with human RBCs were authorized by the ‘Ärztekammer des Saarlandes’ under registration number 132/08 for healthy donors and by the Canton Zürich ethics committee under registration number KEK ZH NR 2010-0237 as well as by the regional ethics committee CPP Sud/Ouest Outre Mer III, Bordeaux under registration number 2010-A00244-35 for SCD patients. In all cases, blood donors provided their written informed consent to participate in this study. This consent procedure was approved by the respective ethics committees under the above-mentioned study registration numbers. For the experiments, we used RBCs from healthy adult donors and non-transfused HbSS SCD patients. Blood was drawn from a vein into heparinized containers and used within 30 h of the withdrawal.

Experiments with mouse blood were carried out in strict accordance with the recommendations in the Guide for the Care and Use of Laboratory Animals of the National Institutes of Health. The protocol was approved by the State Office for Health and Consumer Protection (Permit Number: C1-2.4.3.4). All efforts were made to minimize suffering. Blood samples were collected from the cheeks of the mice by lancet puncture and were collected into heparinized Eppendorf tubes.

The following procedure was followed identically for RBCs from human and mouse. RBCs were isolated via centrifugation at 5000× *g* for 3 min. The buffy coat and plasma were discarded, and the remaining RBCs were washed three times with Tyrode solution (Tyrode) containing the following components (in mM): 135 NaCl, 5.4 KCl, 10 glucose, 1 MgCl_2_, 1.8 CaCl_2_ and 10 HEPES. The pH was adjusted to 7.35 using NaOH. For imaging the cells were loaded with Fluo-4 AM (Molecular Probes, Eugene, OR, USA) at a concentration of 5 μM for 1 h at 37 °C. Then, the cells were washed three times with Tyrode.

### 2.2. Microscopic Video Imaging to Measure Intracellular Ca^2+^

**Figure 1:**
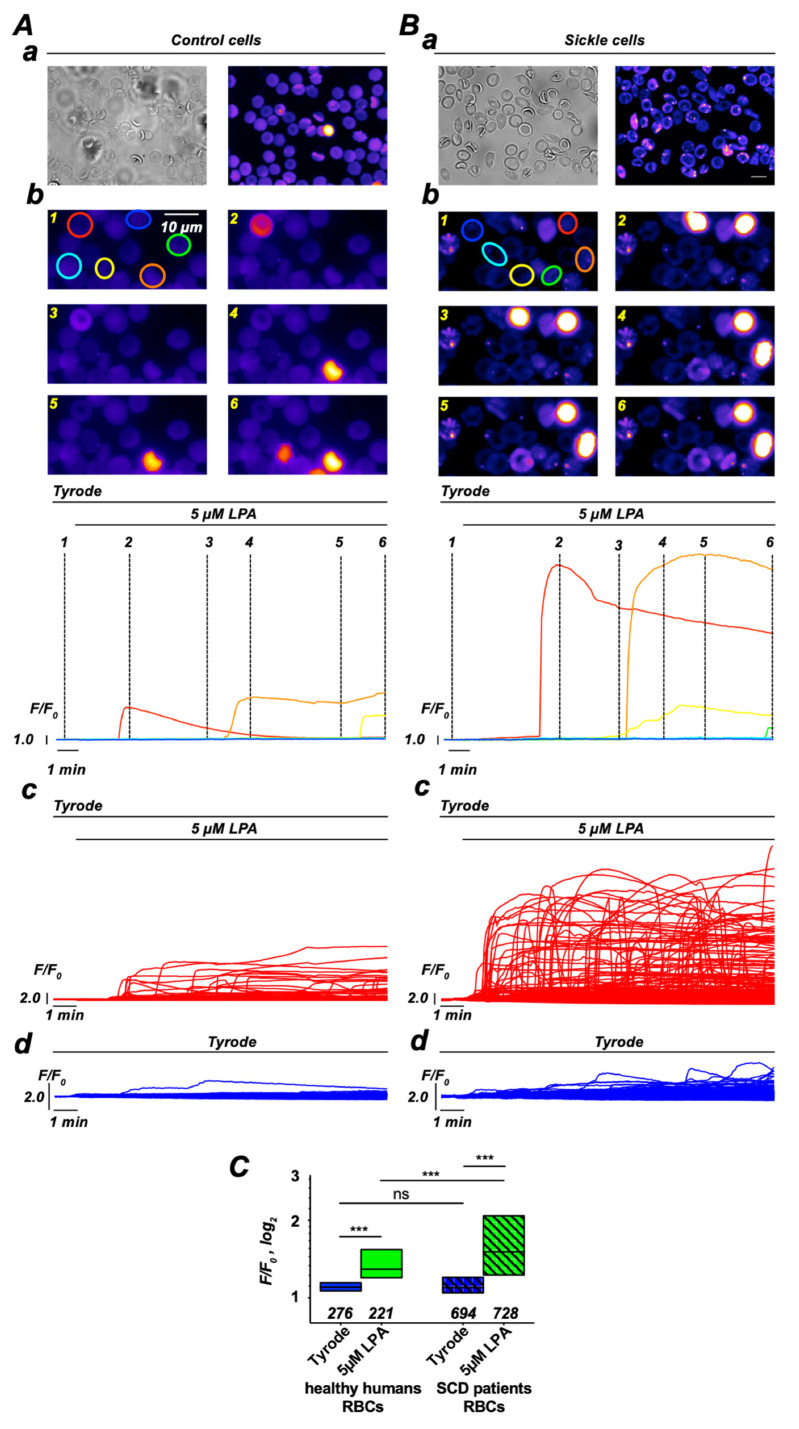
LPA-induced increase in intracellular Ca^2+^ in RBCs from healthy donors compared to HbSS SCD patients. (**A**) depicts the measurements for healthy control cells, while (**B**) shows the same approach for RBCs from SCD patients. (**a**) shows bright field images and fluorescence images under unstimulated control conditions. (**b**) Representative image sections with RBCs marked with regions of interest (ROI) in different colors. In the traces below the images, the fluorescence intensities (selfratio F/F_o_) of the cells marked (same color codes as the ROI) are plotted. The bars above the traces indicate the presence/application of the external solution. Numbers at the dashed lines correspond to the numbers of the images above. (**c**) gives the collection of all fluorescence intensity traces (similar as in (**b**)) but for all cells measured with LPA stimulation. (**d**) shows the same as in (**c**) for control experiments without stimulation. (**C**) Statistical analysis of the max. value of each trace as outlined in (**b**–**d**). Since cellular values are not Gaussian distributed, statistics show medians and 10–90% boxes. Numbers below the boxes refer to the number of cells analyzed originating from at least three different experiments. ns stands for not significant (*p* > 0.05) and *** for *p* < 0.001. All measurements were performed at room temperature.

Life-cell imaging was performed to monitor intracellular Ca^2+^ kinetics in individual cells treated with hormones and pharmaceuticals (see below). Fluo-4-loaded cells were plated onto coverslips. We waited 15 min for cell sedimentation and dye de-esterification. Fluorescence was measured on the stage of an inverted microscope (TE2000, Nikon, Tokyo, Japan) equipped with a 60× Plan Apo 1.4 objective. A video imaging device (TILL Photonics, Munich, Germany) was attached to the microscope and contained a monochromator (Polychrome IV), a camera (Imago), the imaging control unit and acquisition software (TILLvision V4.0) for Figures 1, 3, 4, 6 and 8. The system was upgraded to Polychrome V, a sCMOS camera (Flash4, Hamamatsu Photonics, Hamamamtsu City, Japan) and the software (LightAcquisition, FEI, Munich, Germany) before the experiments presented in Figures 5, 7 and 9 were carried out. Fluo-4-loaded cells were excited at 480 nm, and the resulting fluorescence images (using a 505 nm long pass dichroic mirror and a 535/40 bandpass filter) were collected every 5 s for 15 min. A gravity-driven local perfusion system was utilized to quickly exchange solutions in the field of view. All measurements were performed at room temperature (~22 °C). The images were analyzed in ImageJ (Wayne Rasband, National Institute of Mental Health, Bethesda, MD, USA), and the traces were further processed by IGORpro software (WaveMetrics, Kake Oswego, OR, USA).

### 2.3. Stimulating Substances

All activators and inhibitors were of analytical grade and freshly prepared from frozen stock solution. The stock solution of LPA was 5 mM dissolved in phosphate-buffered saline (PBS, Sigma-Aldrich, St. Louis, MO, USA). Pertussis toxin (PTX, Sigma-Aldrich) was dissolved at a concentration of 250 µg/mL in H_2_O. U0126 as well as Wortmannin (both Sigma-Aldrich) were dissolved at 10 mM in DMSO (Roth, Karlsruhe, Germany). Gö6976 (Tocris Biosciences, Bristol, UK) was dissolved at 1 mM in DMSO and ω-agatoxin-TK (Peptanova, Sandhausen, Germany) at 100 µM in H_2_O. For AlF_4_^−^, 30 µM AlCl_3_ and 10 mM NaF were mixed. FK506 (InvivoGen, Toulouse, France) was dissolved at 20 mM in DMSO and Cyclosporin A (Alfa Aesar, Ward Hill, MA, USA) at 1 mM in ethanol.

### 2.4. Western Blots

For Western blots, RBCs were filtered to make sure samples were not contaminated by mononuclear cells [27]. Briefly, 0.36 g of a mixture of equal parts by weight of microcrystalline cellulose (cod. S5504, Sigma-Aldrich) and α-cellulose (cod. C8002, Sigma-Aldrich) were mixed with 10 mL Tyrode to obtain a homogeneous slurry. The barrel of a 5 mL plastic syringe was used as the filtering device and a septum made of filter paper (Whatman^®^ No 4, Cod. 1004125, Schleicher & Schuell BioScience, Dassel, Germany) was placed on the bottom of the syringe barrel. The cellulose slurry was carefully poured inside the syringe and the cellulose allowed to settle evenly. The excess Tyrode was allowed to flow through, exposing the surface of the cellulose bed. At this point, 1 mL of blood sample was carefully layered on top of the cellulose. When all the blood had entered the cellulose bed, Tyrode was added to the filter, and the fraction of filtered RBCs was collected. The filtered cells were concentrated by centrifugation and washed two more times with Tyrode. HeLa cells used as positive controls were handled as previously described [28].

Cells were lysed by EP extraction buffer (100 mM Tris-HCl, 100 mM NaCl, pH 7.5) supplemented with 0.5% Triton X-100, 20 mM DTT, complete protease inhibitor cocktail and Phospho Stop (Roche Applied Science, Mannheim, Germany) on ice for 30 min. The whole-cell lysates were then centrifuged at 5000× *g* for 10 min. The cell lysate was separated on 12% polyacrylamide gels. The gel electrophoresis was followed by transfer to nitrocellulose transfer membranes (Protran, Whatman, Kent, UK). Blots were incubated with rabbit polyclonal anti-GAPDH (1:15,000), anti-LPAR 1 (1:2000), anti-LPAR 2 (1:1000), anti-LPAR 3 (1:1000, all antibodies LifeSpan BioScience, Seattle, WA, USA), anti-LPAR 4 (1:1000), anti-LPAR 5 (1:1000, both from Aviva Systems Biology, San Diego, CA, USA).

Blots were washed with phosphate-buffered saline with 0.1% tween-20 (PBS-T) and incubated with the horseradish peroxidase (HRP)-conjugated antibodies (1:5000). Enhanced chemiluminescence (ECL) was used for signal detection.

### 2.5. Immunostainings and Confocal Measurements

For LPA receptor immunostainings, RBCs were fixed with 2% PFA plus 0.0075% glutaraldehyde in Tyrode for 30 min. Then cells were permeabilized with 0.3% Triton X-100 in Tyrode for 10 min, followed by centrifugation at 3700× *g* for 5 min. After blocking with 5% bovine serum albumin (BSA) in Tyrode for 20 min, cells were incubated with the first antibody, such as LPAR1-5 (1:50), in blocking buffer (5% BSA in Tyrode) for 4 h at room temperature while gently shaking. After washing in Tyrode3 times, cells were incubated with Alexa Fluor 488 goat anti-rabbit IgG (1:400; Molecular Probes, Eugene, OR, USA) in Tyrode for 2 h. Confocal recordings were performed on a Leica TSC SP5II microscope (Leica, Mannheim, Germany) using a 63× oil immersion objective with a numerical aperture of 1.4 (HCX PL APO) as described recently [29]. Fluorescence images were acquired in the laser scanning mode using Leicas hybrid detector (HyD) at an excitation of 488 nm, and the emission was collected between 491 and 540 nm for the Alexa Fluor secondary antibody. Bright field images were recorded with a Leica DFC290 camera. The Leica Application Suite Advanced Fluorescence (LAS AF) was used for image acquisition while ImageJ (Wayne Rasband, National Institute for Mental Health) was used for image processing, i.e., z-projection of the confocal slices, adjustment of brightness and contrast as well as allocation of a green look-up table.

### 2.6. ELISA Phosphorylation Assay

For the ELISA phosphorylation assay, the RayBio^®^Phospho-Erk1 (T202/Y204)/Erk2 (T185/Y187) ELISA Kit was used to detect MAPK phosphorylation, following the manufacturer’s protocol. In brief, RBCs from healthy donors and SCD patients were treated with Tyrode (control) or 5 µM LPA for 15 min. After RBC lysis using the cell lysate buffer provided in the kit, 100 μL sample or positive control (A431S002-1, provided by kit) to Erk1/2 was placed on a coated microplate. The cell lysates were incubated for 2.5 h at room temperature while shaking. After washing the plates 4 times with the wash solution provided by the kit, they were incubated with 100 μL detection antibody anti Erk1 (T202/Y204)/Erk2 (T185/Y187) for 1 h while shaking. Then 100 μL of prepared 1x HRP-conjugated anti-rabbit IgG was added to each well and incubated for 1 h at room temperature while shaking. After washing another 3 times, 100 μL of TMB one-step substrate reagent was added to each well and incubated for 30 min at room temperature in the dark while shaking. Then 50 μL of stop solution (Item I) was added to each well and read immediately at 450 nm in a PT-FUJI-SCANNER.

### 2.7. Data Analysis

Single-cell traces were analyzed as previously described in detail [30]. All data were tested for Gaussian distribution. If normality was passed, data are presented as the mean values ± SEM in bar graphs. Otherwise, data are presented in box plots. The number under each box plot is the cell number. All experiments were performed on at least 3 different donors/patients or 3 individual mice.

According to the normality test and the grouping conditions for testing significance, Student’s t-test, a Mann–Whitney test, an ordinary one-way ANOVA or a Kruskal–Wallis test with Tukey’s or Dunn multiple comparison test was performed. The level of statistical significance was indicated as *p* < 0.05 (*), *p* < 0.01 (**), *p* < 0.001 (***) and *p* > 0.05 (ns) for not significant. All statistical tests were performed in Prism, GraphPad, La Jolla, CA, USA.

## 3. Results

### 3.1. LPA-Induced Increase in Intracellular Ca^2+^ in RBCs—Comparison of Healthy Donors with SCD Patients

Based on LPA stimulation of single RBCs and the previously introduced fluorescence microscopy analysis method [16,30], we compared Ca^2+^ entry in RBCs of healthy donors and SCD patients (Figure 1). In Figure 1, we provide representative examples of how the analysis was performed from imaging all the way to the statistical analysis. Figure 1A shows representative images and single-cell traces for healthy RBCs; Figure 1B shows the corresponding data for SCD RBCs. The statistical analysis in Figure 1C depicts a significantly higher LPA-induced increase in the intracellular Ca^2+^ in sickle cells compared to healthy RBCs. Please note that also under control conditions we find a small LPA-independent Ca^2+^ entry. Although the topic of this report is LPA-dependent Ca^2+^ entry, this rather small (fractual) Ca^2+^ influx contributes to the entire picture of the Ca^2+^ signaling (see Discussion below).

### 3.2. LPA Receptors in Healthy and SCD RBCs

**Figure 2:**
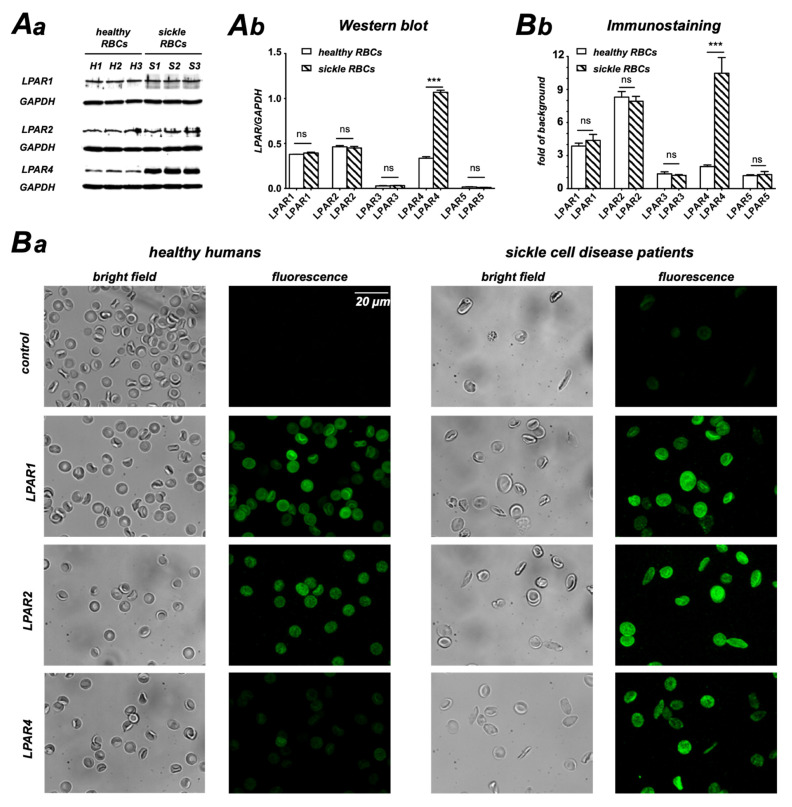
LPA receptors (LPAR) in RBCs from healthy donors compared to SCD patients. (**Aa**) depicts the Western blots of LPAR 1, 2 and 4 for healthy RBCs (H1–H3) and sickle cells (S1–S3). As a loading control, we used GAPDH. (**Ab**) shows the statistical analysis of the Western blots as presented in (**Aa**). For LPARs 3 and 5, the bands were below the detection limit. Examples of full gel Western blots for all LPA receptors tested are provided in Supplemental Figure S1. (**Ba**) shows confocal z-projections of immunocytochemistry staining for control conditions (only secondary Alexa Flour 488-labeled antibody) and for the three abundant LPARs in comparison to brightfield images of healthy (left) and SCD (right) RBCs. Even if the cell number in particular images is rather low, we ensure that they are representative for the cell population. (**Bb**) summarizes the statistical analysis of the immunostainings as depicted in (Ba) supporting the Western blot data. ns stands for not significant (*p* > 0.05) and *** for *p* < 0.001.

Since the Ca^2+^ increase in both healthy and SCD RBCs is caused by the application of LPA, it was a logical step to look at the abundance of LPA receptors in RBCs. Figure 2A shows Western blots and their analysis of the LPA receptors 1–5, which reveal the presence of LPA receptors 1, 2 and 4 in healthy and sickle RBCs, with receptor 4 being significantly increased in SCD RBCs. The immunocytochemistry data and their statistical analysis in Figure 2B clearly support the results of the Western blot. The full gel Western blots for all conditions are provided in Supplemental Figure S1.

### 3.3. G Protein Signaling

**Figure 3:**
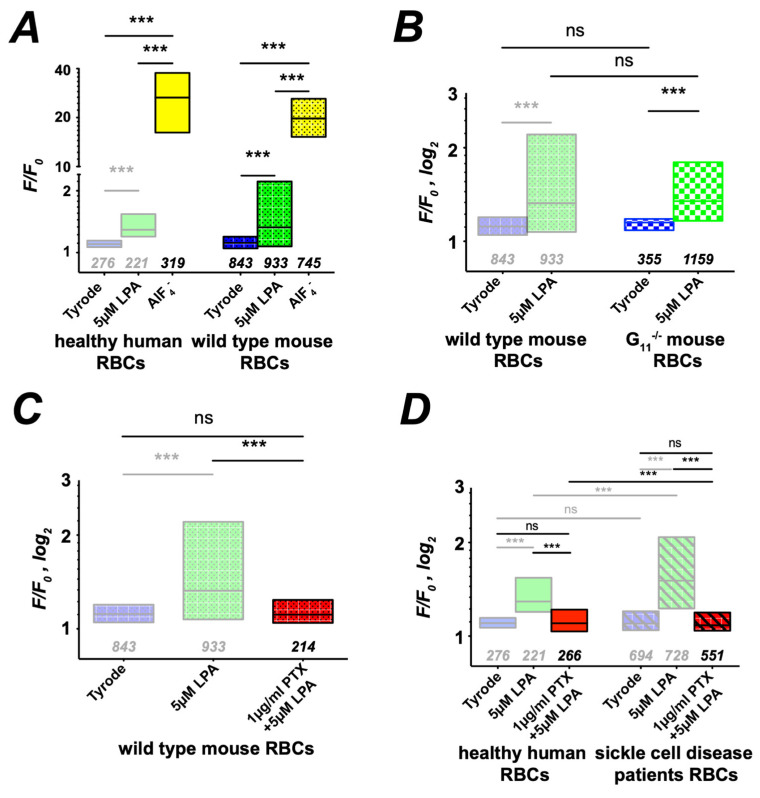
G protein signaling in LPA-induced Ca^2+^ entry into RBCs. More transparent boxes in the statistics indicate data that are replotted for comparison. All panels present the statistical evaluation, with the numbers below the boxes indicating the number of cells measured. (**A**) The pan-specific G protein activator AlF_4_^−^ induces a Ca^2+^ entry in human and mouse RBCs exceeding the one of LPA stimulation. (**B**) LPA-induced Ca^2+^ entry in RBCs of Gα_11_ knock-out mice shows no difference compared to cells of wild-type mice. (**C**) The Gα_i_-specific inhibitor PTX is able to fully block LPA-induced Ca^2+^ entry in mouse RBCs. (**D**) LPA stimulation of PTX-pretreated RBCs of healthy donors and sickle cell disease patients. Numbers below the boxes refer to the number of cells analyzed originating from at least three different experiments. ns stands for not significant (*p* > 0.05) and *** for *p* < 0.001. All measurements were performed at room temperature.

To further explore the molecular signaling pathway, we followed the hints of the literature towards the involvement of G proteins [31] by comparing the LPA stimulation with the broad G protein activation by AlF_4_^−^ [32] in human and mouse RBCs (Figure 3A). Although the magnitudes of the response are slightly different between human and mouse RBCs, both depict a significant increase in intracellular Ca^2+^ even exceeding the LPA stimulation. Following the literature, we probed the LPA stimulation in RBCs of a Gα_11_ knock-out mouse line [33], revealing no difference to cells of wild-type mice (Figure 3B). The next candidate of G proteins to probe was the Gα_i_ utilizing the specific inhibitor PTX [34]. PTX fully inhibits LPA-induced Ca^2+^ entry in mouse RBCs (Figure 3C), leading to the conclusion that the LPA mechanism is exclusively operated by Gα_i_ proteins. The same Gα_i_-based mechanism seems to be operating in human RBCs (Figure 3D).

### 3.4. The PKC Branch of the Signaling Pathway of G Protein Signaling

**Figure 4:**
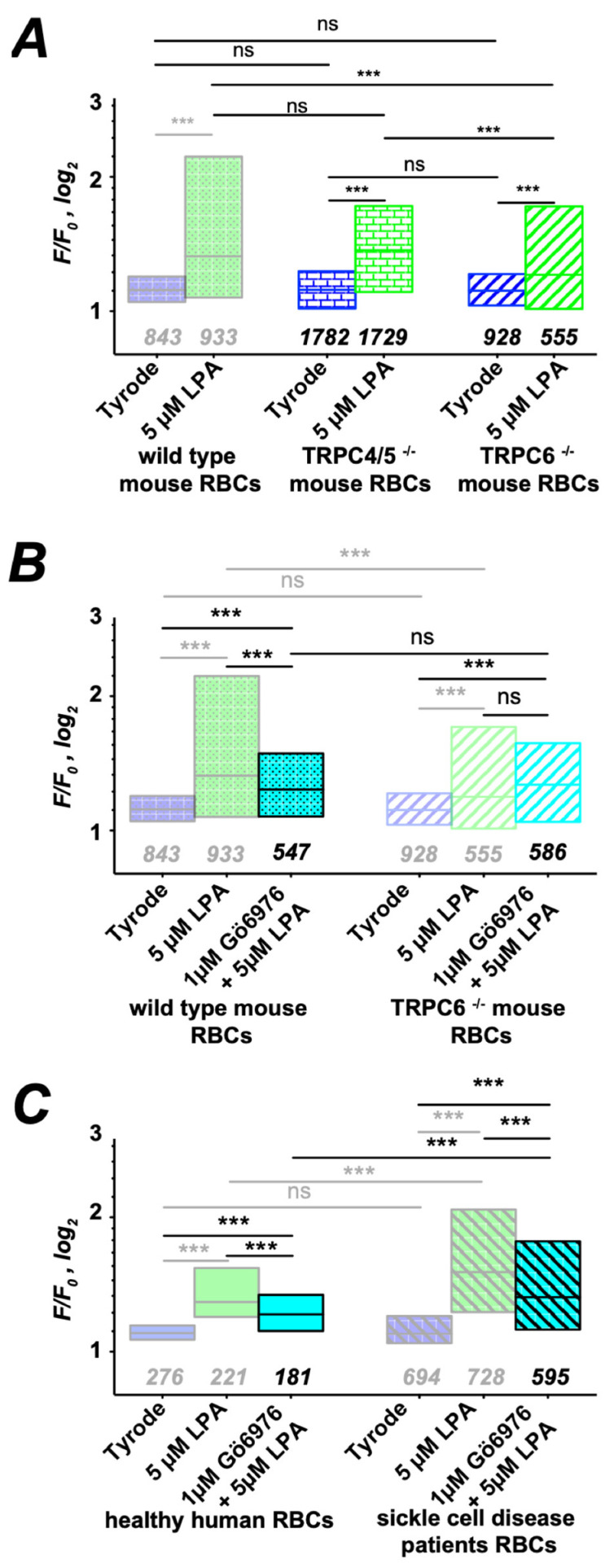
Gα_i_ protein signaling activates TRPC6. More transparent boxes indicate data that are replotted for comparison. All panels present the statistical evaluation, with the numbers below the boxes indicating the number of cells measured. (**A**) LPA stimulation of RBCs from TRPC4/5^−/−^ double knock-out and TRPC6^−/−^ mice in comparison to wild-type mice. (**B**) Gö6976 pretreatment has no effect on the LPA response of TRPC6^−/−^ RBCs but suppresses Ca^2+^ entry in RBCs of wild-type mice. (**C**) Same experiments as in (**B**) but with RBCs from healthy humans and sickle cell disease patients. Numbers below the boxes refer to the number of cells analyzed originating from at least three different experiments. ns stands for not significant (*p* > 0.05) and *** for *p* < 0.001. All measurements were performed at room temperature.

Because Gα_i_ subunits have been associated with the transient receptor potential (TRP) channels C4 and C5 [35], we investigated RBCs fromTRPC4/5 double knock-out mice [36]. As shown in Figure 4A, there is no significant difference in Ca^2+^ entry of TRPC4/5^−/−^ RBCs compared to WT RBCs. Because TRPC6 has been reported to contribute to the increase in residual Ca^2+^ influx in RBC [37], we further investigated RBCs of TRPC6^−/−^ mice [36,37]. The stimulation with LPA revealed that Ca^2+^ entry in TRPC6^−/−^ RBCs is suppressed, but still significantly increased compared to control conditions (Figure 4A). Due to the proposed involvement of PKCα in RBCs channel regulation [18,24,38], we inhibited PKCα with the specific inhibitor Gö6976 [39]. The results are depicted in Figure 4B. In RBCs of wild-type mice, inhibition of PKCα lead to a decrease in Ca^2+^ influx, which is still higher than that under control conditions. For TRPC6^−/−^ mice, Gö6976 had no additional effect on LPA-induced Ca^2+^ entry. In human RBCs (both healthy and SCD), the relative amount of Gö6976-inhibited Ca^2+^ entry is identical compared to wild-type mouse conditions (Figure 4C). In addition, we considered the application of TRPC6 inhibitors, in particular larixyl acetate [40] and SAR7334 [41] at the suggested concentrations. However, as depicted in Supplemental Figure S2, both inhibitors show non-specific side effects that result rather in a Ca^2+^ increase instead of an inhibition. This is clearly seen when applied to RBCs of TRPC6^−/−^ mice.

**Figure 5:**
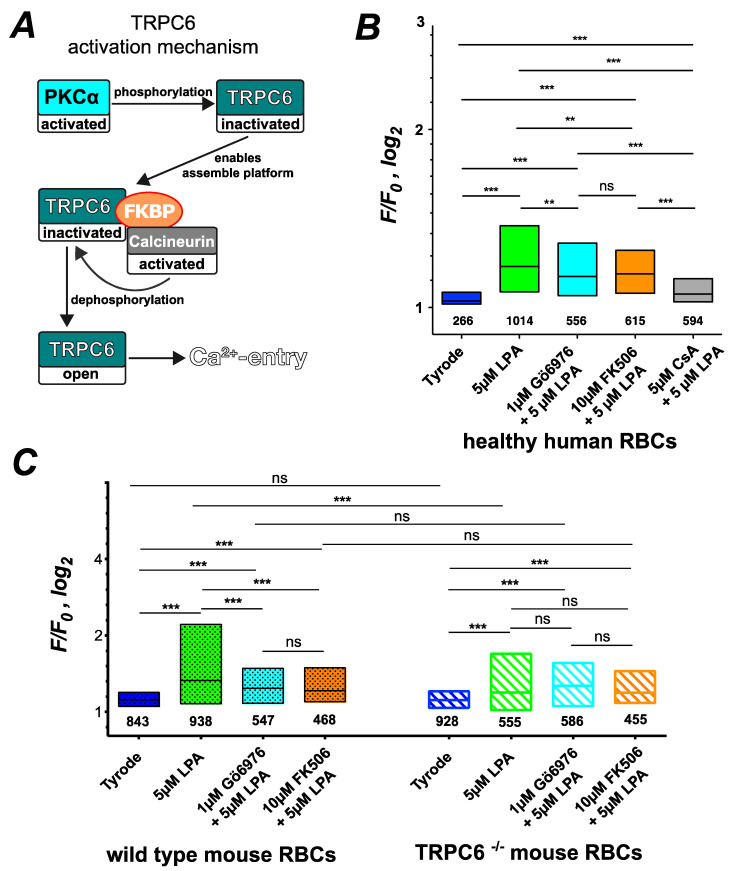
Activation mechanism of TRPC6 by PKCα. (**A**) shows a scheme of the TRPC6 activation mechanism by PKCα. (**B**) presents the statistical evaluation of human RBCs pretreated with Gö6976, FK506 or Cyclosporin A (CsA) followed by LPA stimulation. (**C**) depicts the statistical evaluation of wild-type mouse RBCs in comparison to RBCs from TRPC6^−/−^ mice for perfusion with Tyrode (control), 5 µM LPA stimulation and 5 µM LPA stimulation with pretreatment of 1 µM Gö6976 or 10 µM FK506. The numbers below the boxes indicate the number of cells measured in at least three different experiments. ns stands for not significant (*p* > 0.05), ** for *p* < 0.01 and *** for *p* < 0.001. All measurements were performed at room temperature.

As shown in Figure 4, we could link PKCα activity to TRPC6-mediated Ca^2+^ entry. We were puzzled by this finding, because phosphorylation of TRPC6 by PKC isoforms is commonly associated with the inhibition of TRPC6 [42]. However, Kim and Saffen proposed that the PKC-induced phosphorylation of TRPC6 and its inactivation enable the binding of the immunophilin FKBP12 to TRPC6, eventually forming a multiprotein complex with calcineurin and calmodulin [43]. The calcineurin then dephosphorylates TRPC6 and in turn activates the channel [43]. This interaction scheme is sketched in Figure 5A. To test whether this mechanism might be responsible for PKCα-induced TRPC6 activity in RBCs, we compared LPA-induced Ca^2+^ influx of human RBCs under Gö6976 pretreatment with that of RBCs pretreated with FKBP12 inhibitor FK506 and the calcineurin inhibitor Cyclosporin A (CsA) (Figure 5B). FK506 suppresses LPA-induced Ca^2+^ entry to exactly the same amount as Gö6976 (Figure 5B), supporting the mechanism sketched in Figure 5A. Inhibition of LPA-induced Ca^2+^ entry by CsA is slightly more enhanced than by Gö6976 or FK506 (Figure 5B), supporting the mechanism depicted in Figure 5A further but indicating a crosstalk of CsA with the PI3 kinase-mediated branch of the signaling pathway (see below). Additionally, we tested the effect of FK506 in comparison to Gö6976 treatment in mouse RBCs and TRPC6^−/−^ mouse RBCs (Figure 5C). In both cases, there was no difference between the Gö6976 treatment and that with FK506. Furthermore, for TRPC6^−/−^ RBCs, Gö6976 and FK506, application lacked a significant difference to pure stimulation with 5 µM LPA, providing further support for the mechanism presented in Figure 5A.

### 3.5. The MAP Kinase Branch of the Signaling Pathway

**Figure 6:**
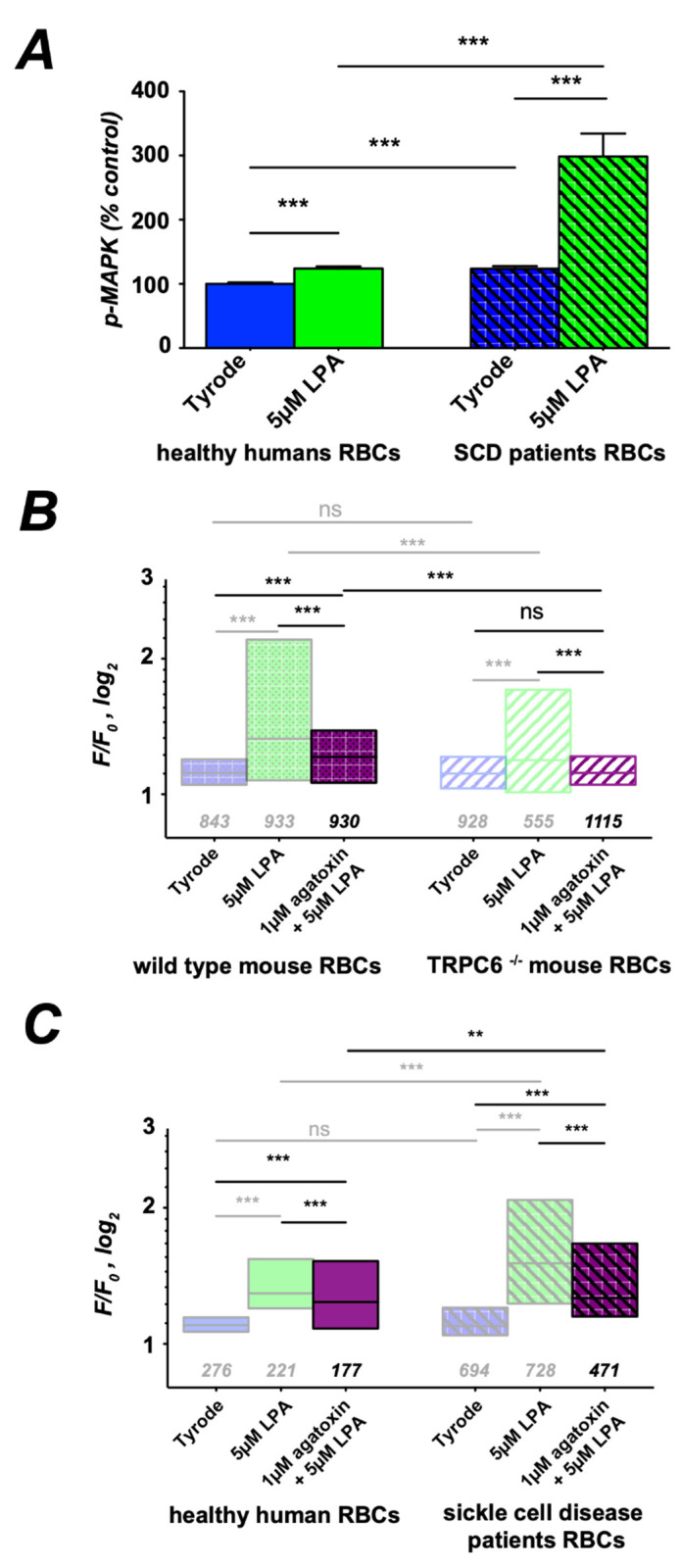
A Gα_i_ protein singling activates MAPK and ω-agatoxin-TK-sensitive Ca^2+^ entry. (**A**) Phosphorylation assay for MAPK activity by ELISA for RBCs from healthy humans and sickle cell disease patients at rest (Tyrode) and after 15 min of 5 µM LPA stimulation. (**B**,**C**) More transparent boxes in the statistics indicate data that are replotted for comparison. (**B**,**C**) present the statistical evaluation, with the numbers below the boxes indicating the number of cells measured. (**B**) LPA stimulation of RBCs from wild-type and TRPC6^−/−^ mice with and without ω-agatoxin-TK pretreatment was followed by fluorescence read out of the Ca^2+^ fluorophore Fluo-4. (**C**) Same experiments as in (**B**) but with RBCs from healthy humans and SCD patients. All measurements were performed at room temperature. ns stands for not significant (*p* > 0.05), ** for *p* < 0.01 and *** for *p* < 0.001. All measurements were performed at room temperature.

In search of the cause of the remaining amount of Ca^2+^ entry, we focused on a MAP kinase (MAPK) pathway which was reported to be abundant in RBCs [44]. Therefore, we probed RBCs from healthy donors and SCD patients for MAPK activity using an ELISA. Figure 6A depicts the results revealing an increased basal MAPK activity in SCD RBCs.

LPA induces an increase in MAPK activity, which is more pronounced in RBCs from SCD patients. The Ca^2+^ channel that is regulated by this signaling pathway is expected to be ω-agatoxin-TK sensitive [17,24]. Based on the pharmacology and Western blots of the α_1A_ subunit, this channel was proposed to be Ca_V_2.1 [17,18]. We performed the Ca^2+^-imaging assay of LPA stimulation with and without pre-incubation of ω-agatoxin-TK on RBCs of WT mice and TRPC6^−/−^ mice (Figure 6B). ω-agatoxin-TK partly inhibits LPA-induced Ca^2+^ entry in RBCs from WT mice, whereas in RBCs of TRPC6^−/−^ mice, ω-agatoxin-TK keeps internal Ca^2+^ levels the same as under control conditions.

In human RBCs, Ca^2+^ entry upon incubation with ω-agatoxin-TK (Figure 6C) is qualitatively the same as in wild-type mouse RBCs (Figure 6B). In RBCs of SCD patients, the Ca^2+^ response to LPA upon incubation with ω-agatoxin-TK is increased compared to healthy donors (Figure 6C).

**Figure 7:**
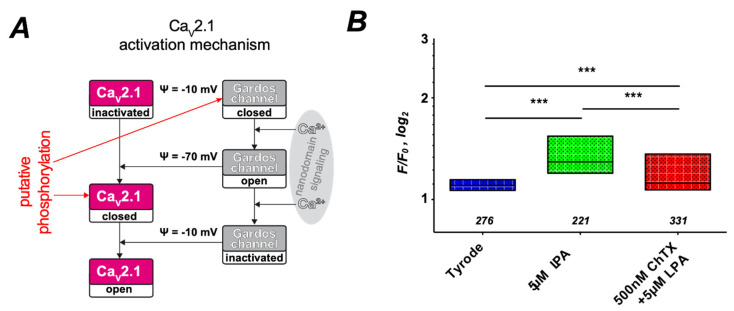
Activation mechanism of Ca_V_2.1 by the Gárdos channel. (**A**) shows a scheme of the Ca_V_2.1 activation mechanism involving the Gárdos channel. (**B**) presents the statistical evaluation of RBCs pretreated with the Gárdos channel inhibitor Charybdotoxin (ChTX) followed by LPA stimulation, with the numbers below the boxes indicating the number of cells measured in at least three different experiments. *** stands for *p* < 0.001. All measurements were performed at room temperature.

The results presented in Figure 6B,C point to an involvement of the Ca_V_2.1 channel that was shown to be present in RBCs [18]. On first view, it seems odd that a voltage-gated channel could be activated in non-excitable RBCs. However, we have developed a concept that is based on the activation of the Gárdos channel (Ca^2+^-activated K^+^ channel, KCNN4, K_Ca_3.1), which leads to RBC hyperpolarization from −10 mV to approximately −70 mV [45]. We recently provided experimental evidence for this hypothesis [46]. The main idea is summarized in Figure 7A.

The data presented in Figure 6 indicate that kinase activity favors the channel opening. It is unclear whether the Gárdos channel or Ca_V_2.1 is phosphorylated. Both channels contain numerous putative phosphorylation sites. Although we kept both options in the scheme of Figure 7A, we favor the phosphorylation of the Gárdos channel, which makes more sense conceptionally and was also reported to occur in neurons [47].

**Figure 8:**
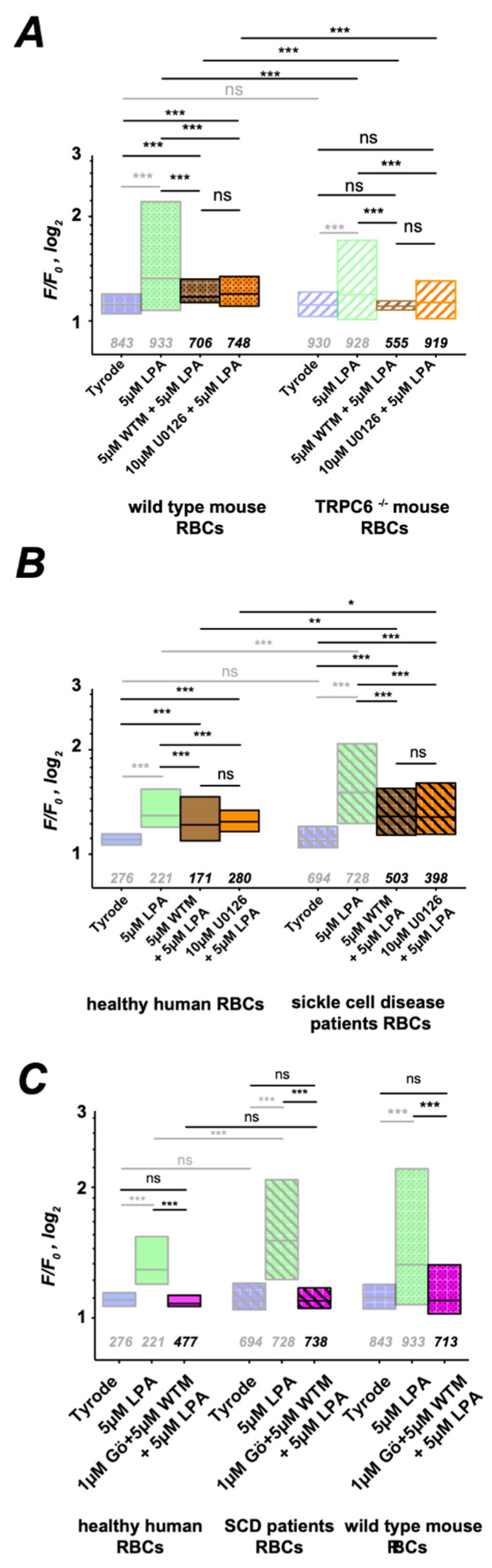
The MAPK-associated pathway. More transparent boxes in the statistics indicate data that are replotted for comparison. All panels present the statistical evaluation, with the numbers below the boxes indicating the number of cells measured. (**A**) LPA stimulation of RBCs from wild-type and TRPC6^−/−^ mice after pretreatment with Wortmannin or U0126 was followed by fluorescence read out of the Ca^2+^ fluorophore Fluo-4. (**B**) Same experiments as in (**A**) but with RBCs from healthy humans and sickle cell disease patients. (**C**) shows the statistical evaluation of RBCs pretreated with Gö6976 and Wortmannin followed by LPA stimulation. All measurements were performed at room temperature. ns stands for not significant (*p* > 0.05) * for *p* < 0.05, ** for *p* < 0.01 and *** for *p* < 0.001.

If indeed the Gárdos channel is involved in the activation of Ca_V_2.1, inhibition of the Gárdos channel should have an impact on LPA-induced Ca^2+^ entry. The result of this experiment is depicted in Figure 7B and indeed shows a significantly lower Ca^2+^ level when the LPA stimulation is performed in the presence of 500 nM of the Gárdos channel inhibitor Charybdotoxin (ChTX). To complement the approach presented in Figure 6, we performed further pharmacological investigations and pre-incubated the RBCs with U0126, an inhibitor of MEK [48], the upstream activator of MAPK, as well as with Wortmannin (WTM) a phosphoinositide 3 kinase (PI3 kinase) inhibitor [49]. Both substances showed basically the same effect (Figure 8). Figure 8A reveals a suppression of LPA-induced Ca^2+^ entry by the pharmacological interventions in wild-type RBCs, while in TRPC6^−/−^ RBCs, Ca^2+^ entry is completely abolished. In human RBCs, Ca^2+^ entry upon incubation with the blocking agents (Figure 8B) is qualitatively the same as in wild-type mouse RBCs (Figure 8A). In RBCs of SCD patients, the Ca^2+^ response to LPA upon incubation with WTM and U0126 is upscaled compared to healthy donors (Figure 8B).

### 3.6. The Overview of the Signaling Cascade

After identifying the two signaling branches, the remaining question is whether the two branches completely account for the total LPA-induced Ca^2+^ entry in RBCs. The experiments with the TRPC6^−/−^ mouse RBCs in Figure 6B and Figure 8A already point in this direction. The experiments depicted in Figure 8C provide further evidence that the two branches of the pathway, the PKCα mediated (blocked by Gö6976) and the PI3 kinase activated (blocked by WTM), can completely explain LPA-induced Ca^2+^ entry into RBCs of healthy humans, sickle cell disease patients and mice.

**Figure 9:**
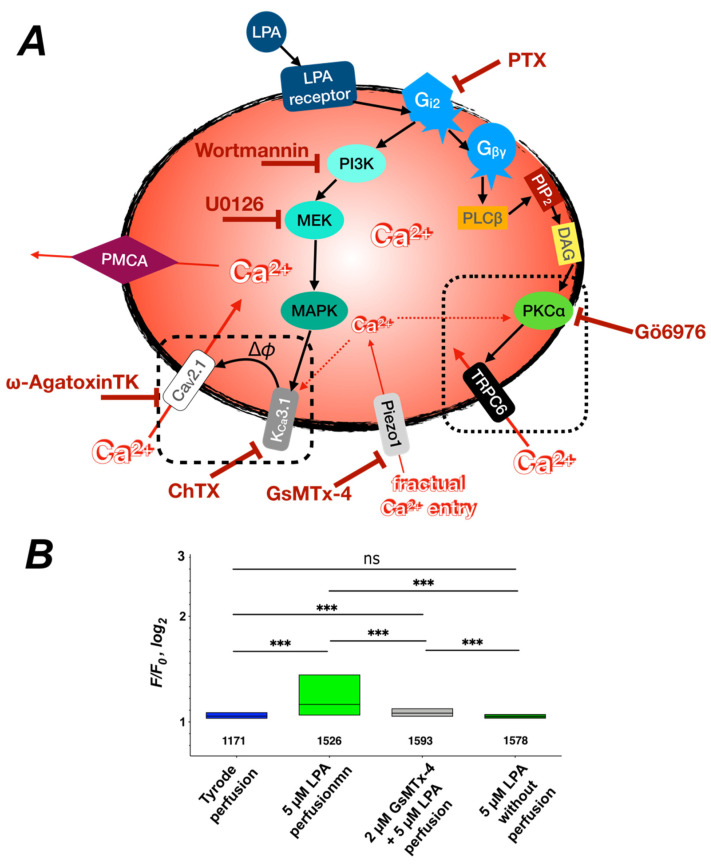
Overview of the Ca^2+^ signaling cascade. (**A**) shows a scheme of the hypothesized Ca^2+^ signaling cascade based on the indications presented in this paper. It includes the pharmacological tools (inhibitors) used to probe the RBCs (dark red). The cascade starts with LPA stimulation of LPA receptors as outlined in Figure 1 and Figure 2. G protein signaling was shown in Figure 3. The involvement of PKCα and TRPC6 in one of the signaling branches was indicated in Figure 4. PKCα is generally activated through the involvement of phospholipase C_β_ (PLCβ), phosphatidylinositol-4,5-bisphosphate (PIP_2_) and diacylglycerol (DAG) [56]. The detailed activation of TRPC6 by PKCα (black dotted box) is shown in Figure 5A. The second signaling branch involving PI3 kinase (PI3K), MEP, MAP kinase (MAPK) and Ca_V_2.1 was explored in Figure 6, Figure 7 and Figure 8, whereas the detailed interaction between Ca_V_2.1 and the Gárdos channel (black dashed box) is detailed in Figure 7A. Indications for a fractual Ca^2+^ signaling by a mechanosensitive ion channel are provided in (**B**). Piezo1 as indicated by the scheme is only an example for a mechanosensitive channel. The plasma membrane Ca^2+^ pump (PMCA) was not investigated within this paper but since it is the major transport mechanism of Ca^2+^ out of the RBC, it was added to complete the overall picture. (**B**) presents the statistical evaluation of human RBCs pretreated with the inhibitor of mechanosensitive channels, GsMTx-4, followed by LPA stimulation, with the numbers below the boxes indicating the number of cells measured in at least three different experiments. In contrast to all other experiments, for the measurement of the grey bar, no local perfusion system was used to completely avoid any shear stress to the cells. ns stands for *p* < 0.05 and *** for *p* < 0.001. All measurements were performed at room temperature.

A schematic overview of the signaling cascade is given in Figure 9A. Here it becomes evident that both branches of the signaling cascade require Ca^2+^ to be activated. The PKC branch needs Ca^2+^ to activate PKCα [50] and the MAP kinase branch requires Ca^2+^ to activate the Gárdos channel [11]. One could argue that both pathways stimulate each other by positive feedback loops, but also one branch operates if the other one is inhibited, as shown in Figure 6B and Figure 8.

Both in vivo as well as in our in vitro experiments, RBCs experience a certain shear stress. In vivo, this is almost obvious and there it results in a measurable Ca^2+^ increase [51] most probably mediated by the mechanosensitive channel Piezo1, which is abundant in RBCs [52,53,54]. In our in vitro experiments, we used a gravity-driven local perfusion system, which results in a very moderate shear stress applied to the cells in the field of view in the microscope, which is much below physiological levels (0.3–10 Pa) in vivo. However, this flow/shear stress occurs in all experiments also under control conditions, when only Tyrode is perfused. Having a closer look at these control experiments (Figure 1Ad,C), the Ca^2+^ does not remain constant (self-ratio F/F_o_ of 1) but slightly increases. Therefore, a small fractional Ca^2+^ entry by a mechanosensitive channel such as Piezo1 could be the Ca^2+^ source enabling both branches of the signaling cascade.

To test this hypothesis, we performed experiments on human RBCs, where the 5 µM LPA were applied (i) without perfusion (less exact time point of application) and (ii) in the presence of 2 µM of the mechanosensitive channel inhibitor GsMTx-4 [55]. The results are presented in Figure 9B and reveal that indeed LPA-induced Ca^2+^ entry could be prevented under both additional conditions tested, supporting the hypothesis that a mechanosensitive channel such as Piezo1 provides fractional Ca^2+^ entry, enabling the signaling cascades as outlined in Figure 9A.

## 4. Discussion

When judging the results, we like to point out that RBCs are very special cells and although much of our current knowledge of membrane transport originates from RBCs when they have been ‘the’ model cell for membrane transport during the last century [57], they lack a number of mechanisms. First of all, there is the lack of organelles in mammalian RBCs, i.e., they do not contain cell internal ion stores, a property which favored them as model cells. Consequently, well-known processes such as Ca^2+^-induced Ca^2+^ release or store-operated Ca^2+^ channels do not exist in RBCs. Furthermore, it is worthwhile to mention that mature mammalian RBCs do not contain translational machinery, i.e., they have the set of proteins from when the erythroblasts enucleated and the RBCs were released into the circulation. Depending on the aging process of the particular proteins, young and old RBCs contain different sets of proteins [58], i.e., receptor, ion channel and kinase content, just to name a few proteins, vary during the RBC lifetime of in average approximately 115 days in humans [59] and 45 days in mice [60]. Additionally, the number of ion channel copies is very low in RBCs—for the Gárdos channel, an estimated 1–5 copies per cell in 75% of the cells [61,62], and Piezo1 and TRPC6 are also within the detection limit [63]. Therefore, functional measurements, especially when they integrate the signal as in Ca^2+^-imaging experiments, are more sensitive than molecular biological approaches.

### 4.1. Elucidating the Signaling Mechanism of LPA-Induced Ca^2+^ Entry in RBCs

Our results show that Ca^2+^ uptake evoked by LPA stimulation and well described in healthy RBCs [4,6,17,21,30] is markedly increased in SCD (Figure 1). As the molecular mechanisms of LPA-induced Ca^2+^ influx are so far unknown, we aimed at elucidating the various steps in the signaling cascade in healthy RBCs and compared it with pathological RBCs. By using Western blot and immunocytochemistry, we first probed for LPA receptors and found that whereas the same isoforms (1,2 and 4) were expressed in both populations, LPA receptor type 4 was augmented more than 3-fold in SCD RBCs (Figure 2). The increase in Ca^2+^ influx in both cell populations was conveyed by two branches of a signaling pathway starting at an LPA receptor and the further activation of a Gα_i_ protein (Figure 3). It is worthwhile to mention that full activation of G proteins by AlF_4_^−^ exceeds by far the Gα_i_ activation by LPA receptors (Figure 3A), confirming the abundance of various G proteins in RBCs [64]. One branch of the signaling pathway regulated the TRPC6 channel through recruitment of PKCα (Figure 4 and Figure 5), whereas the other points to the opening of the Ca_V_2.1 channel involving PI3, MEK and MAP kinases and the stochastic activity of the Gárdos channel (Figure 6, Figure 7 and Figure 8). The suggested signaling is heavily based on experimental evidence from experiments with knock-out mice (Figure 3, Figure 4, Figure 5, Figure 6, Figure 7 and Figure 8). Although this genetic-based approach cannot be directly transferred to human RBCs, a number of pharmacological studies, which show a high functional homology between mouse and human RBCs (Figure 3, Figure 4, Figure 5, Figure 6, Figure 7 and Figure 8), suggest a projection of the signaling cascade (Figure 9A) to humans.

### 4.2. LPA Signaling in RBCs of Sickle Cell Disease Patients

The cause of the increased Ca^2+^ response of SCD RBCs presumably is at least partially the LPA receptor 4, whose abundance compared to healthy cells acts as an amplifying switch for the entire signaling cascade. We hypothesize that the increased density of particular receptors might be a general feature of sickle cells. This notion is supported by the fact that NMDA receptors also show a higher abundance in sickle cells compared to healthy RBCs [65]. One of the reasons behind this resides in the average age of SCD RBCs (average lifetime of 10–20 days [66]) compared to healthy RBCs (average lifetime of approximately 115 days [59]), and since RBCs do not produce new proteins, receptors are lost over time.

The LPA signaling pathway and the consecutive activation of channels as well as the discovery of NMDA receptors in RBCs [13] place Ca^2+^ channels in focus as pharmaceutical targets for the treatment of one of the most severe symptoms of SCD, i.e., VOC. This holds particular true, as Ca^2+^ has been identified as a trigger for RBC self-aggregation [6,22,67] and for their adhesion to endothelial cells [68,69,70].

### 4.3. Potential for Heterogeneity

Recently, direct channel activation of LPA in RBC was discussed [4,6]. However, tremendous heterogeneity in the LPA response of the RBCs of each donor [16,17,30] (cp. also Figure 1Ac,Bc) could not be comprehensively explained by RBC age [30,71]. In light of the current results revealing a substantial signaling cascade with numerous directly involved molecular players, this provides a potential chain where each individual link may cause a modulation of the final Ca^2+^ signal. This variety of direct players is supplemented by a presumably varying contribution of indirect modulators, particularly the plasma membrane Ca^2+^ ATPase (PMCA) [72], which is activated by 60–100 nM Ca^2+^ [73] and may directly pump out the Ca^2+^ which enters the RBCs, and its activity will depend on the metabolic status of the RBC, which can vary from cell to cell.

### 4.4. Novel Molecular Interactions

Although the abundance of Gα_i_ protein is well documented in RBCs [74,75], this is, to our knowledge, the first report of a Gα_i_–PKCα interaction in RBCs. However, for platelets, such a signaling step has been previously reported [76]. Data on the interaction of PKC and TRPC6 are rather scarce and mostly show suppression of TRPC6 expression by PKC [77,78], which is irrelevant to the fast changes induced by PKCα in the TRPC6 channel in RBCs, leading to an acute increase in the Ca^2+^ influx within minutes. Direct interaction (phosphorylation) of TRPC6 by PKC is still controversial [42,79,80]. However, relying on single-channel recordings, in coronary artery smooth muscle cells, an inhibition of TRPC6 by inhibition of PKC was reported [81]—similar to what we found in RBCs.

Activation of a PI3 kinase/MAP kinase pathway by Gα_i_ has already been described in the context of platelets [82,83]. The pharmacological inhibition of LPA-induced Ca^2+^ entry in TRPC6^−/−^ RBCs using ω-agatoxin-TK (Figure 6B) provides clear evidence for the involvement of Ca_V_2.1 in the proposed signaling cascade. Although the modulation of voltage-activated Ca^2+^ channels by G protein-induced signaling has been reported [84], the abundance and operation of voltage-gated channels in non-excitable cells is anything but established. Even though such discussions were performed in the context of T lymphocytes, where the expression and function of voltage-gated Ca^2+^ channels have been shown [85,86,87], our interpretation follows the idea of hyperpolarization–depolarization jumps caused by fluctuations in Gárdos channel activity. This hypothesis was previously published [45], and recently supported with experimental evidence [46]. In the present paper, we were able to add experimental evidence: when the K^+^-conducting Gárdos channel was blocked, LPA-induced Ca^2+^ entry was also diminished (Figure 7B)—to a similar extent as observed with modulation by the Ca_V_2.1 channel inhibitor ω-agatoxin-TK (Figure 6C). We have to admit that this concept only works because (i) the number of Gárdos channels in RBCs is very low [61,62] and (ii) we face the situation of a submaximal Gárdos channel activation. As a consequence the probability of the Gárdos channels opening leads to stochastic opening and closing, which enables membrane potential flickering. Such depolarization–hyperpolarization cycles are required for the activation of a voltage-gated channel.

## 5. Conclusions

We found a new Ca^2+^ signaling cascade that is increased in RBCs of SCD patients in the first place by an increased occurrence of the LPA receptor 4. However, since G_i_ protein signaling triggers an entire signaling cascade, we provide a set of new pharmacological targets that might be promising in addressing the most severe symptom of SCD, i.e., VOC.

## Data Availability

The data presented in this study are available on request from the corresponding author. The data are not publicly available due to privacy restrictions.

## Supplementary Materials

The following are available online at https://www.mdpi.com/2073-4409/10/2/456/s1, Figure S1: Gel electrophoresis analysis of LPA receptors 1–5 in human red blood cells (RBCs) of healthy donors and sickle cell disease (SCD) patients; Figure S2: Testing the reported TRPC6 inhibitors larixyl acetate and SAR7334.
