# Supplementary material for: Lysophosphatidic Acid-Activated Calcium Signaling Is Elevated in Red Cells from Sickle Cell Disease Patients
Source: Cells. 2021 Feb 20;10(2):456. doi: 10.3390/cells10020456 (PMC7924404; doi:10.3390/cells10020456)
Supplement: Supplementary file 1 [file cells-10-00456-s001.pdf]

Lysophosphatidic acid activated Calcium signaling is elevated in red cells from sickle cell disease patients

Jue Wang [1], Laura Hertz [2,3], Sandra Ruppenthal [3,4], Wassim El Nemer [5,6], Philippe Connes [7], Jeroen S. Goede [8], Anna Bogdanova [9], Lutz Birnbaumer [10,11], and Lars Kaestner [2,3]*

[1] Department of Cellular and Molecular Biology, The University of Texas Health Science Center at Tyler, Tyler, Texas, United States of America; anjwan@googlemail.com
[2] Theoretical Medicine and Biosciences, Saarland University, 66421 Homburg, Germany; laurahertz@gmx.de
[3] Experimental Physics, Dynamics of Fluids, Saarland University, 66123 Saarbrücken, Germany; lars_kaestner@me.com
[4] Gynaecology, Obstetrics and Reproductive Medicine, Saarland University Hospital, 66421 Homburg, Germany; SLRuppenthal@gmx.de
[5] Etablissement Français du Sang PACA-Corse, Marseille, France; Aix Marseille Univ, EFS, CNRS, ADES, "Biologie des Groupes Sanguins", Marseille, France; wassim.el-nemer@efs.sante.fr
[6] Laboratoire d'Excellence GR-Ex, Paris, France
[7] Laboratory LIBM EA7424, Vascular Biology and Red Blood Cell teal, University Claude Bernard Lyon 1, 69008 Lyon, France; pconnes@yahoo.fr
[8] Division of Oncology and Hematology, Kantonsspital Winterthur, CH-8401 Winterthur, Switzerland; Jeroen.goede@ksw.ch
[9] Red Blood Cell Research Group, Institute of Veterinary Physiology, University of Zürich, CH-8057 Zürich, Switzerland; annab@access.uzh.ch
[10] Institute of Biomedical Research (BIOMED), Catholic University of Argentina, C1107AFF Buenos Aires, Argentina
[11] Laboratory of Neurobiology, National Institute of Environmental Health Sciences, Research Triangle Park, North Carolina 27709, USA; birnbau1@gmail.com

Supplemental Material

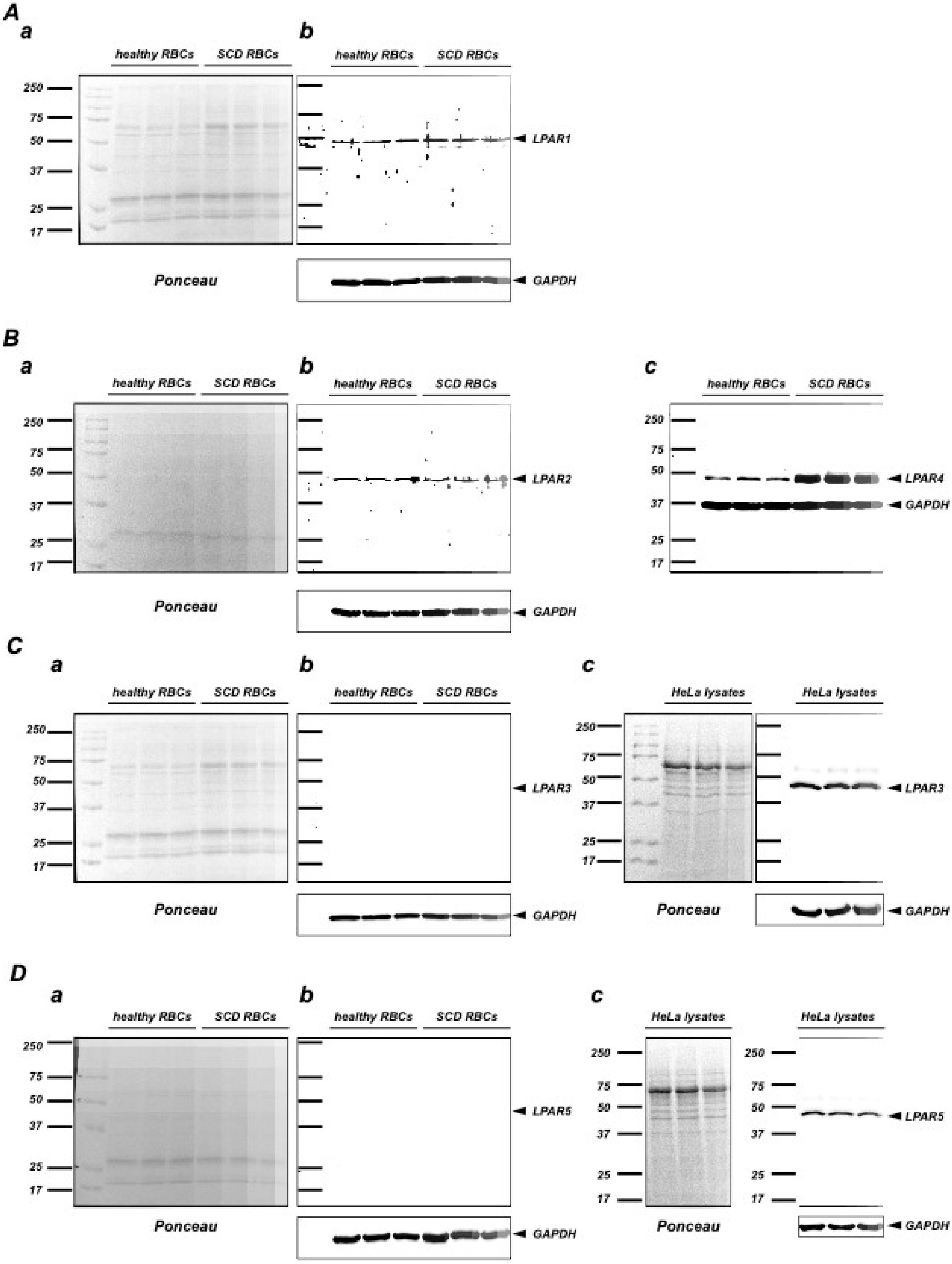

Supplemental Figure S1: Gel electrophoresis analysis of LPA-receptors 1-5 in human red blood cells (RBCs) of healthy donors and sickle cell disease (SCD) patients. (**A**) Analysis of LPA-receptor 1 (LPAR1). (**Aa**) Ponceau stained gel to see the total protein distribution. (**Ab**) Western blot of the LPAR1 (for methodological details see main document). The GAPDH was used for normalization of the quantitative analysis (compare Figure 2A of the main document). GAPDH is plotted separately because it required a different exposure time than LPAR1. (**B**) Analysis of LPA-receptor 2 (LPAR2) and LPA-receptor 4 (LPAR4). (**Ba**) Ponceau stained gel to see the total protein distribution. The same gel was used for both Western blots (LPAR2 and LPAR4). (**Bb**) Western blot of the LPAR2 (for methodological details

see main document). The GAPDH was used for normalization of the quantitative analysis (compare Figure 2A of the main document). GAPDH is plotted separately because it required a different exposure time than LPAR2. (**Bc**) Western blot of the LPAR4. Here GAPDH could be visualized in the same blot. (**C**) Analysis of LPA-receptor 3 (LPAR3). (**Ca**) Ponceau stained gel to see the total protein distribution. (**Cb**) Western blot of the LPAR3 (for methodological details see main document). The GAPDH was used for normalization of the quantitative analysis (compare Figure 2A of the main document). GAPDH is plotted separately because it was recorded at a different exposure time than LPAR3. (**Cc**) Since the Western blot of LPAR3 did not show detectable bands for RBCs, a positive control was performed with HeLa cells. The Poncaeu staining as well as the Western blot of LPAR3 are depicted. GAPDH is plotted separately because it required a different exposure time than LPAR3. (**D**) Analysis of LPA-receptor 5 (LPAR5). (**Da**) Ponceau stained gel to see the total protein distribution. (**Db**) Western blot of the LPAR5 (for methodological details see main document). The GAPDH was used for normalization of the quantitative analysis (compare Figure 2A of the main document). GAPDH is plotted separately because it was recorded at a different exposure time than LPAR5. (**Dc**) Since the Western blot of LPAR5 did not show detectable bands for RBCs, a positive control was performed with HeLa cells. The Poncaeu staining as well as the Western blot of LPAR5 are depicted. GAPDH is plotted separately because it required a different exposure time than LPAR5.

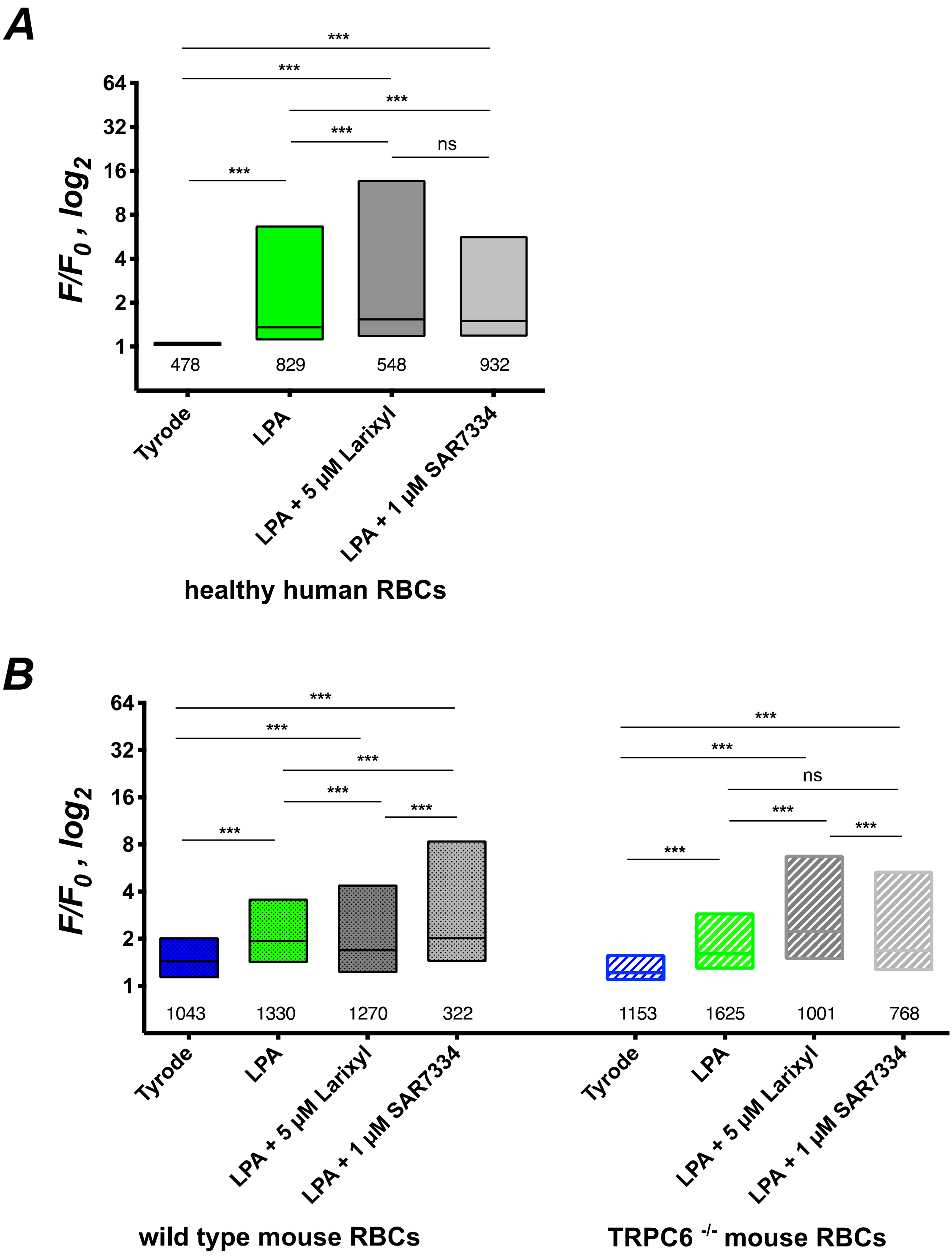

Supplemental Figure S2: Test of the reported TRPC6 inhibitors larixyl acetate and SAR7334. (**A**) Statistical evaluation of human RBCs pretreated with the TRPC6 inhibitors larixyl acetate (Larixyl) and SAR7334 followed by LPA stimulation. (**B**) Statistical evaluation of wild type mouse RBCs in comparison to RBC from TRPC6$^{-/-}$ mice for perfusion with Tyrode (control), LPA stimulation and LPA stimulation with pretreatment of 5 µM Laryxil or 1 µM SAR7334. The numbers below the boxes indicate the number of cells measured in at least 3 different experiments. ns stands for not significant ($p>0.05$) and *** for $p<0.001$. All measurements were performed at room temperature.
